# Biomimetic and personalized nanovaccines in cancer immunotherapy: Design innovations, translational challenges, and future directions

**DOI:** 10.1016/j.jare.2026.01.070

**Published:** 2026-02-02

**Authors:** Suling Xu, Haitao Yang, Boris Minev, Wenxue Ma

**Affiliations:** a Department of Dermatology, The First Affiliated Hospital of Ningbo University, Ningbo 315010, China; b Center for Gene and Cell Therapy Program, Southern University of Science and Technology, Shenzhen, Guangdong 518055, China; c CureScience Institute, San Diego, CA 92121, USA; d Department of Radiation Medicine and Applied Sciences, University of California, San Diego, CA 92093, USA; e Sanford Stem Cell Institute, Department of Medicine, and Moores Cancer Center, University of California San Diego, La Jolla, CA 92093, USA

**Keywords:** Nanovaccines, Biomimetic nanoparticles, Personalized cancer vaccines, Neoantigen, Cancer immunotherapy, mRNA vaccines

## Abstract

Personalized nanovaccines represent advancing frontier in cancer immunotherapy by integrating patient-specific tumor antigens with biomimetic delivery systems to enhance immune activation, targeting precision and clinical efficacy. Biomimetic platforms including exosome-, red blood cell (RBC)-, and immune cell membrane-coated nanoparticles, as well as artificial antigen-presenting cell (aAPC) systems, recreate native biological interfaces to improve antigen presentation, macrophage polarization, and adaptive immune priming. Despite significant progress, major translational challenges remain, including incomplete mechanistic understanding, regulatory complexity, and manufacturing scalability. This review synthesizes recent advances in biomimetic and personalized nanovaccine design, highlighting clinical progress in lipid nanoparticle (LNP)-based vaccines such as BNT111 and mRNA-4157, emerging innate immune adjuvants including Toll-like receptor (TLR) and stimulator of interferon genes (STING) agonists, and rational combination strategies with immune checkpoint blockade. Key safety and quality consideration including immunotoxicity, off-target immune activation, and batch heterogeneity are critically evaluated alongside emerging engineering solutions. Finally, future directions integrating AI-guided neoantigen prediction, modular microfluidic manufacturing, and multi-omic biomarker frameworks are discussed to accelerate next generation cancer nanovaccine translation.

## Highlights

• Biomimetic nanovaccines enhance tumor-specific antigen delivery and adaptive immune priming.
• RBC, exosome and immune cell membrane coatings improve circulation longevity and targeting.
• Personalized mRNA and peptide nanovaccines demonstrate synergy with immune checkpoint blockade.
• GMP manufacturing, regulatory complexity, and safety profiling remain key translational barriers.
• AI-guided neoantigen design and modular and microfluidics support scalable personalized vaccine production.

## Graphical abstract

**Figure:**
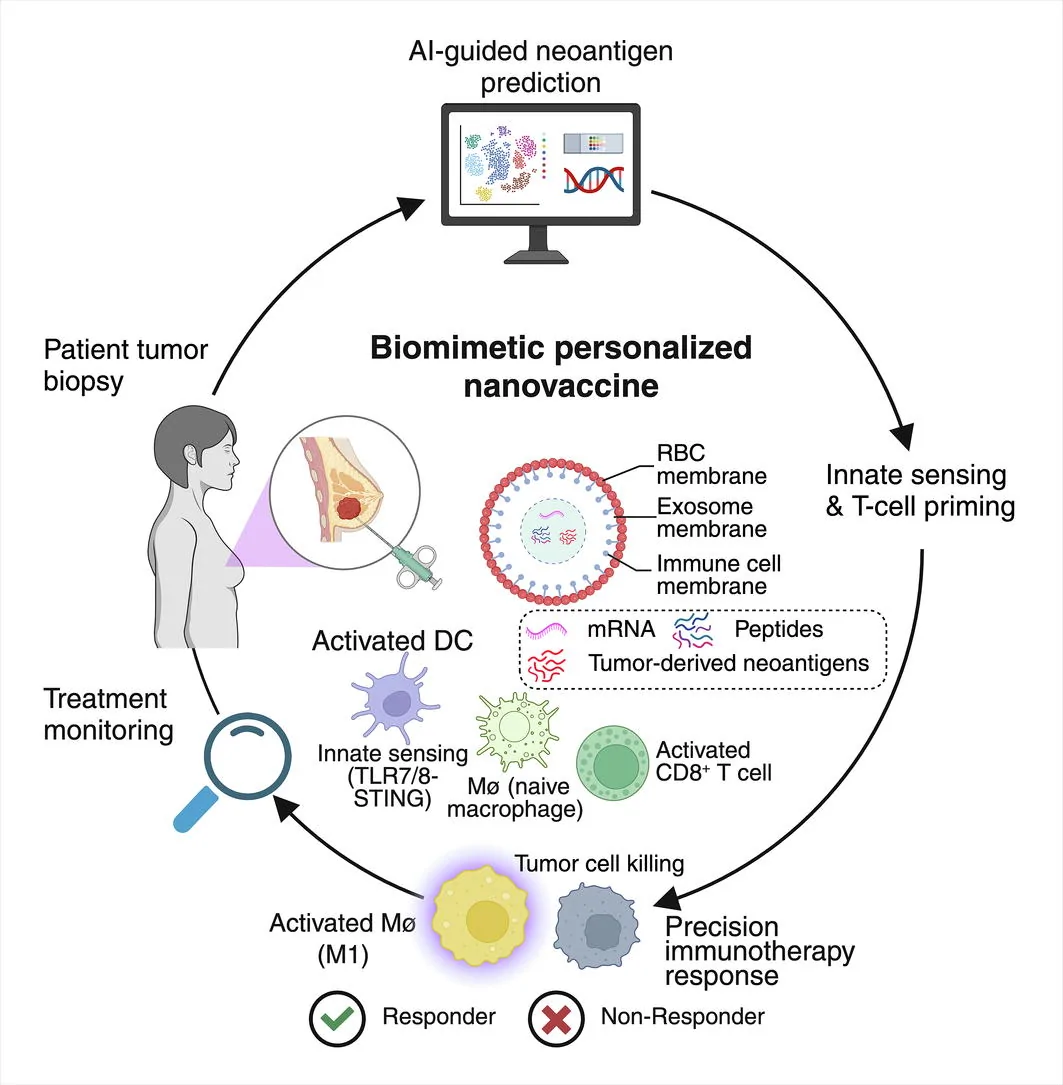

## Introduction

Cancer vaccines, particularly peptide- and mRNA-based platforms have emerged as promising immunotherapeutic strategies by eliciting antigen-specific immune responses that prime cytotoxic T cells against tumors [1]. Despite this promise, their clinical efficacy remains limited due to several biological and translational challenges. These include rapid antigen degradation, inefficient intracellular delivery, limited trafficking to lymph nodes, and insufficient immune activation [2], [3]. Although nanotechnology has advanced the delivery and protection of tumor antigens, many conventional nanocarriers are hindered by poor biocompatibility, rapid systemic clearance, and inadequate tumor specificity, limiting their broader clinical application [4], [5].

Despite notable progress, critical gaps remain in optimizing immune targeting, clinical scalability, and long-term efficacy. A persistent challenge lies in integrating biomimetic delivery mechanisms with personalized antigen payloads and combination immunotherapy. This review was motivated by the urgent need to synthesize these evolving strategies into a unified translational framework for next-generation cancer nanovaccine design.

While prior reviews have focused on isolated aspects such as neoantigen discovery, nanocarrier chemistry, or immune adjuvant selection, few have comprehensively examined how biomimetic coatings such as red blood cell (RBC) membranes, exosomes, macrophage membranes, and artificial antigen-presenting cells (aAPCs) can be strategically coupled with personalized payloads (e.g., tumor–derived mRNA or peptide libraries) to enhance immune evasion, prolong systemic circulation, and improve lymphatic trafficking [6], [7], [8]. Furthermore, such payloads can be customized to reflect the patient's tumor-specific mutanome, supporting precision immunotherapy that moves beyond “one-size-fits-all” vaccination paradigms [9], [10], [11].

In parallel, combination regimens such as immune checkpoint inhibitors (ICIs), stimulator of interferon genes (STING), or toll-like receptor (TLR) agonists, and radiotherapy can be co-delivered or sequentially integrated to further boost antigen presentation, dendritic cell (DC) activation, and T cell expansion [12], [13]. However, their integration of these combination strategies into nanovaccine platforms remains underdeveloped in both preclinical studies and clinical translation [14], [15].

Building on our prior work in nanovaccine development and translational cancer immunotherapy [16], [17], [18], [19], [20], [21], [22], [23], we aim to address key unmet challenges. This review outlines how biomimetic coatings, personalized payloads, and combination immunotherapies can be rationally integrated into nanovaccine systems to improve antigen specificity, therapeutic efficacy, and clinical applicability [9], [24]. We begin with a concise historical overview of cancer vaccine evolution, followed by an in-depth analysis of design principles linking delivery efficiency with antigen personalization. Finally, we examine major translational bottlenecks, including good manufacturing practice (GMP) compliance, regulatory challenges, and interpatient variability, and explore how emerging technologies such as artificial intelligence (AI), systems biology, synthetic biology and modular microfluidic manufacturing platforms may enable smart, automated, automated, and scalable production of next-generation cancer nanovaccines.

## Transition from conventional to nanotechnology-enabled cancer vaccines

Historically, cancer vaccines have relied on conventional platforms such as whole tumor lysates, inactivated tumor cells, or adjuvanted peptides/proteins [2], [25]. Although these approaches have generated sporadic clinical benefit, they typically exhibit low immunogenicity, inefficient delivery to antigen-presenting cells (APCs), and limited capacity for patient-specific antigen presentation [26], [27]. These limitations, combined with the rapid evolution of tumor immune evasion mechanisms, have driven the field toward more advanced delivery technologies.

The emergence of nanotechnology-enabled vaccine platforms marks a transformative shift in cancer vaccine design [25]. Engineered nanoparticles offer several key advantages over traditional formulations, including protection of labile mRNA or peptide antigens, enhanced cellular uptake by DCs, improved lymph node trafficking, and programmable antigen release kinetics [28], [29]. A particularly powerful advancement is the use of biomimetic coatings, in which nanoparticles are cloaked with natural or synthetic membrane materials. These coatings including red blood cell (RBC) membranes, exosomes, immune-cell membranes, cancer-associated fibroblast (CAF) membranes, and virus-like particles (VLPs) replicate key features of native cellular surfaces to improve circulation time, enable immune evasion*,* and enhance APC targeting [28], [30].

To contextualize the mechanistic concepts introduced in this section, Fig. 1 provides an integrated schematic of biomimetic nanovaccine function. The figure outlines the major membrane-coated nanoparticle platforms (Panel A), their binding, uptake, and intracellular routing by distinct APC subsets (Panel B), and the downstream antigen presentation pathways that drive CD4^+^ and CD8^+^ T-cell activation (Panel C). Together, these visual elements illustrate how membrane composition and nanoparticle architecture influence immune engagement, thereby establishing the conceptual framework for the platform-specific mechanisms discussed in section 3.

**Fig. 1:**
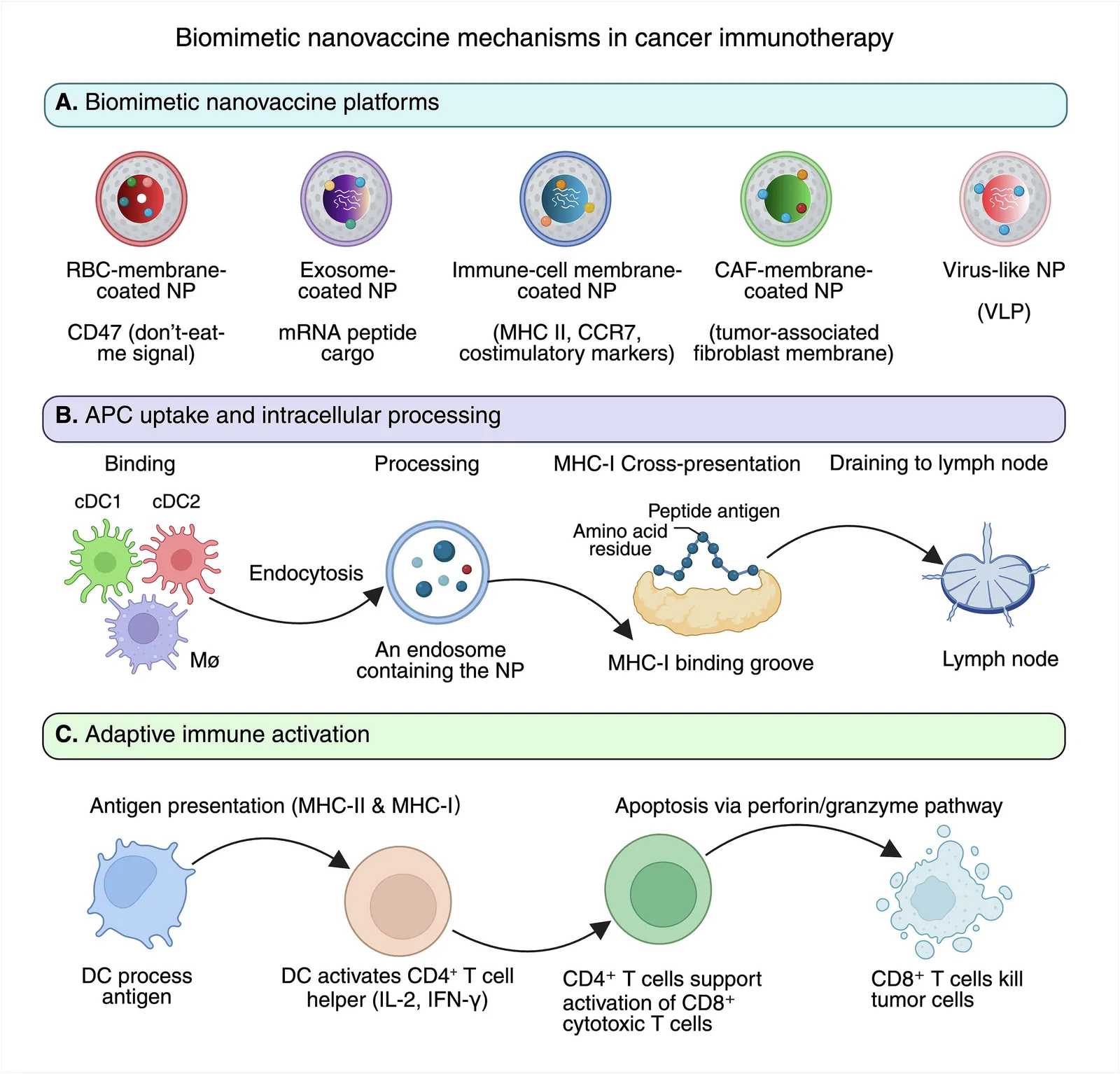
**Biomimetic nanovaccine mechanisms in cancer immunotherapy.** (A) Representative biomimetic nanovaccine platforms, including RBC-membrane-coated nanoparticles, exosome-coated nanoparticles, immune-cell-membrane-coated nanoparticles, CAF-membrane-coated nanoparticles, and virus-like particles. These coatings contribute distinct biological features such as immune evasion signals, endogenous antigenic cargo, or tumor-associated membrane components. (B) APC subtype-specific uptake and intracellular processing of biomimetic nanoparticles. cDC1, cDC2, and macrophages bind and internalize nanoparticles through endocytosis, followed by endosomal trafficking and partial endosomal escape of antigenic cargo. Processed peptides are subsequently loaded onto the MHC-I binding groove for cross-presentation and transported to draining lymph node. (C) Adaptive immune activation. Presented antigens stimulate CD4^+^ helper T cells and CD8^+^ cytotoxic T cells through MHC-II and MHC-I antigen presentation, respectively. Activated CD4^+^ T cells provide cytokine support (e.g., IL-2, IFN-γ) that facilitates CD8^+^ T-cell clonal expansion. CD8^+^ effector T cells then mediate tumor-cell killing through perforin- and granzyme-dependent apoptosis.

Despite the rapid progression of nanotechnology-enabled cancer vaccines, significant translational hurdles remain [25], [31], [32]. The key challenges include optimal integration of precise immunostimulatory cues with delivery vehicles, rational personalization based on patient-specific neoantigens, challenges in large-scale GMP manufacturing, and the need regulatory frameworks capable of accommodating complex hybrid biologic-nanomaterial systems [30], [33], [34]. Addressing these obstacles will be essential for realizing the full clinical potential of biomimetic nanovaccines.

## Biomimetic nanoparticle coatings for cancer vaccines

Biomimetic membrane coatings derived from natural cell type or engineered analogs, offer a compelling strategy to improve the safety, stability, and immunogenicity of nanoparticle-based cancer vaccines [35], [36]. By recapitulating features of native cell surface, these coatings improve immune evasion, prolong blood circulation time, and facilitate targeted delivery to lymphoid tissues where antigen presentation and T-cell priming occurs [37]. Depending on their biological origin, such as RBCs, exosomes, or immune cells, cancer-associated fibroblasts (CAFs), or synthetic membranes, biomimetic coatings provide distinct immunological and pharmacokinetic advantages that can be tuned independently of the antigen payload [38], [39]. The following subsection describes the mechanistic benefits, current applications and translational considerations for major biomimetic coating platforms.

### RBC membrane-coated nanoparticles

RBC membrane-coated nanoparticles leverage the intrinsic immuno-evasive properties of erythrocytes to improve vaccine delivery and systemic compatibility [40], [41], [42]. Human RBCs circulate for approximately 120 days naturally escape phagocytic clearance due to the absence of major histocompatibility complex (MHC) molecules and the presence of self-recognition signals such as CD47, which transmits a “don’t eat me” signal to macrophages [43], [44]. Cloakng nanoparticles with RBC membranes transfers to the nanocarriers, enhancing circulation half-life and reducing unintended innate immune activation [45], [46].

#### Mechanisms of immune evasion and prolonged circulation

The RBC membrane forms a native lipid bilayer enriched with membrane proteins such as CD47, band 3 (also known as anion exchanger 1, AE1), and glycophorins that collectively suppress opsonization, inhibit complement activation, and reduce phagocytic uptake by the reticuloendothelial system (RES) [47], [48]. As a result, RBC-coated nanoparticles display pharmacokinetics and improved biodistribution to secondary lymphoid organs such as the spleen and lymph nodes [49], [50], [51], [52]. These features are especially advantageous for mRNA or peptide vaccines that require timely interaction with APCs before degradation [53].

#### Advantages in cancer vaccine delivery

RBC membrane-coating offer several key advantages for cancer vaccine applications [41]. First, the native lipid bilayers provide a protective effective cargo protection, stabilizing encapsulated mRNA or peptide antigens against enzymatic degradation by serum nucleases and proteases in the bloodstream [54]. In addition, RBC membranes exhibit excellent biocompatibility, as both autologous and allogeneic sources are well tolerated across autologous and allogeneic sources, supporting repeated dosing without inducing significant immunogenicity [55], [56]. The nano-scale dimensions and surface features of the RBC membrane-coated nanoparticles facilitate efficient DC uptake and promote accumulation within lymph nodes, thereby improving antigen processing and T-cell immune priming [28], [57]. Finally, RBC membranes are compatible with various nanoparticle cores, including lipid nanoparticles (LNPs), poly(lactic-co-glycolic acid) (PLGA) particles, and inorganic nanocarriers, providing broad platform flexibility for mRNA and peptide vaccine modalities [58].

#### Current applications in mRNA and peptide nanovaccines

Although extensively studied for drug delivery, applications of RBC membrane-coated nanovaccines for mRNA and peptide cancer vaccines are still emerging [40], [59], [60]. For mRNA delivery, hybrid RBC-LNP platforms have improved mRNA protection, extended antigen expression, and enhance DC uptake [61], [62]. Similarly, RBC membrane-coated PLGA nanoparticles loaded with tumor-associated peptides have demonstrated improved serum stability and superior cytotoxic T lymphocyte (CTL) responses in preclinical tumor models [4], [63], [64]. Immunogenicity can be further boosted by incorporating TLR agonists or immunostimulatory adjuvants within the nanocarriers to enhance cross-presentation and boot CD8^+^ T cell activation [65], [66]. To further enhance immunogenicity, hybrid RBC-cancer cell membrane strategies are also being explored to co-deliver personalized antigenic signatures and immunomodulatory cues, offering a promising direction for personalized neoantigen nanovaccines [15], [25], [30].

### Exosome-coated nanoparticles

Exosomes are nanoscale extracellular vesicles (30–150 nm) naturally secreted by cells and capable transferring mRNA, proteins, lipids and regulatory RNAs, enabling efficient intercellular communication [67], [68], [69]. Their native biocompatibility, complex membrane protein composition, and intrinsic immunomodulatory make them highly attractive candidates for coating nanoparticle platforms aimed at enhancing vaccine targeting and delivery [70], [71]. However, clinical translation is hampered by donor heterogeneity, purification complexity, and GMP standardization remain major barriers for clinical translation.

#### Natural cargo delivery and immune targeting

Exosome membranes naturally carry diverse biological cargos including mRNA, peptides, proteins, and small RNAs and possess adhesion molecules such as integrins and tetraspanins that promote selective uptake by APCs, particularly DCs [33], [70], [72], [73], [74], [75], [76]. These membrane components enhance nanocarrier internalization and support efficient antigen cross-presentation by facilitating receptor-mediated binding and endocytic trafficking.

In addition to their intrinsic uptake properties, exosome-coated nanoparticles preserve the tissue tropism of their donor cells. Tumor-derived exosomes (TEXs) preferentially home to their tissue of origin or draining lymph nodes, whereas DC-derived exosomes (Dexs) carry MHC-peptide complexes and co-stimulatory molecules that can directly prime adaptive immune responses without requiring live DCs [68], [77], [78], [79]. This origin-specific natural targeting capability enables precise payload delivery within the tumor microenvironment (TME) and secondary lymphoid tissues, thereby enhancing the potency and functional breadth of nanovaccine-induced antitumor immunity [80].

#### Sources of exosomes: tumor, DC, and engineered cell lines

Exosomes used for biomimetic nanoparticle coating can originate from diverse donor cell types, each imparting distinct immunological properties and therapeutic roles [81]. TEXs carry a broad repertoire of tumor-associated antigens (TAAs), neoantigens, and nucleic-acid cargo that mirror the molecular profile of the parental tumor, enabling individualized antigen presentation [82]. However, TEXs may also contain immunosuppressive molecules such as PD-L1, TGF-β, and regulatory miRNAs, which can dampen antitumor immunity and therefore require pairing with immune adjuvants or co-stimulatory signals for optimal vaccine performance [68], [80].

In contrast, DC-derived exosomes (Dexs) are inherently immunostimulatory, enriched in MHC-peptide complexes, CD80 and CD86 co-stimulatory molecules, and adhesion molecules that directly activate both CD4^+^ and CD8^+^ T cells without requiring live DCs [76], [83], [84]. These features make Dex-coated nanoparticles particularly effective at enhancing antigen cross-presentation and expanding cytotoxic T-cell responses.

Engineered exosomes generated from mesenchymal stem cells or immortalized producer cell lines provide scalable production, customizable membrane composition, and improved manufacturing consistency, enabling precise modulation of immunological features and facilitating more reproducible biomimetic nanovaccine platforms suitable for clinical translation [68], [85].

#### Challenges in clinical translation

Clinical challenges of exosome-coated nanovaccines is constrained by the inherent heterogeneity of exosomes, which may vary widely in size, cargo composition, and membrane protein profiles depending on the source cell type, physiological state, and isolation method [86], [87]. This variability complicates batch-to-batch reproducibility and contributes to the difficulty achieving GMP-grade purity and manufacturing consistency. Current purification techniques such as ultracentrifugation, size-exclusion chromatography, and microfluidic separation differ substantially in yield, efficiency, scalability and purity, and may co-isolate non-functional vesicles or protein contaminants that reduce biological activity [85].

Immunological ambiguity presents an additional challenge: while some exosomes promote antitumor immunity, others, particularly TEXs may carry immunosuppressive molecules such as programmed death-ligand 1 (PD-L1), transforming growth factor beta (TGF-β), or regulatory miRNAs that blunt effective T-cell responses [79], [88]. To overcome these challenges, engineered exosomes-liposome hybrids, genetically modified donor cells systems, and adjuvant-integrated formulations are being developed to enhance antigen presentation, standardize membrane composition, and improve immunostimulatory potency [89], [90].

### Artificial and emerging biomimetic membrane-coated nanoparticles

Artificial membrane-coated nanoparticles represent a fully engineered alternative to natural biological coatings, offering precise control over lipid composition, ligand density, immunomodulatory, and scalable GMP-compatible manufacturing [91], [92]. This tunability allows artificial membranes to be optimized for antigen loading, endosomal escape, APC targeting, or co-delivery of adjuvants, thereby supporting more predictable pharmacokinetics and immune activation profiles.

In parallel, several emerging natural membrane platforms including macrophage-derived membranes, cancer-associated fibroblast (CAF) membranes, and virus-like particles (VLPs) have expanded the functional repertoire of biomimetic nanovaccines [46], [81], [93], [94], [95]. These coatings retain native chemokine receptors, adhesion molecules, and structural motifs that facilitate tumor homing, DC engagement, and efficient lymph-node trafficking. By mimicking key biological interfaces, these natural systems complement artificial membranes and collectively enhance antigen delivery, cross-presentation, and T-cell priming.

#### Synthetic lipid bilayers and polymer-based coatings

Artificial coatings commonly employ phospholipid bilayers (e.g., liposomes) or amphiphilic polymers such as PEGylated lipids, poloxamers, or polypeptides that protect encapsulated cargo, support dendritic-cell targeting, and enable programmable immune interactions [96]. These engineered coatings form stable vesicles that encapsulate mRNA or peptide antigens from enzymatic degradation while permitting surface functionalization with targeting ligands (e.g., mannose for DCs), TLR agonists, or immune-checkpoint modulators to enhance APC uptake and shape downstream antigen presentation [97], [98], [99], [100].

Recent advances have introduced pH-sensitive, enzyme-responsive, or redox-responsive linkers that allow controlled antigen release within the acidic or enzyme-rich TME, thereby improving therapeutic precision and minimize off-target toxicity [101], [102].

#### Functionalization with immune ligands or tumor antigens

Artificial membranes can be functionalized to present TAAs, neoepitopes, co-stimulatory molecules, or checkpoint-modulating molecules [103]. Presenting MHC-binding epitopes or co-stimulatory ligands directly on the nanoparticle surface enables these platforms to function as synthetic antigen-presenting cells, enhancing recognition and activation of antigen-specific T cells [104], [105].

Incorporation of immunostimulatory adjuvants such as CpG oligonucleotides or monophosphoryl lipid A (MPLA) further activate innate immune activation and supports efficient cross-priming by DCs [106], [107]. Moreover, embedding immune checkpoint modulators, such as anti-PD-1 peptides or CD47 mimetics helps overcome local immunosuppression and amplify vaccine efficacy [44], [108]. These modular design strategies make artificial membranes highly adaptable for both prophylactic and therapeutic cancer vaccination settings.

#### Integration of emerging natural biomimetic platforms

Biomimetic hybrid nanovaccines have recently emerged as a versatile class of platforms that integrate two or more membrane sources such as RBC-exosome hybrids, tumor-RBC composites, or immune cell membrane-polymer assemblies to combine complementary biological functions within a single system. Hybridization enables synergistic performance: RBC-derived membranes contribute long-circulation and immune-evasion properties, whereas exosomal or tumor-derived components introduce adhesion molecules, costimulatory proteins, and tumor-associated antigens that enhance APC uptake and cross-presentation efficiency [109], [110], [111]. In parallel, membrane-polymer hybrid vesicles improve membrane stability, allow tunable incorporation of adjuvants or mRNA cargo, and offer better control over nanoparticle stiffness and degradation kinetics, thereby addressing major limitations of fully biological membranes [111], [112]. Taken together, these hybrid biomimetic systems demonstrate superior lymph-node trafficking, stronger T-cell priming, and enhanced intratumoral accumulation compared with single-membrane platforms, reinforcing their growing importance in next-generation cancer nanovaccine design [113].

#### Opportunities and current applications

Artificial and emerging biomimetic systems have demonstrated strong preclinical efficacy in delivering peptide and mRNA vaccines across melanoma, breast cancer, glioblastoma models [30], [60]. Their capacity to combine antigen delivery, immune activation, and tissue targeting within a single platform positions them highly attractive candidates for rational combination immunotherapies [35], [46], [60]. Hybrid platforms such as lipid-polymer hybrid nanoparticles (LPHNs), further enhance vaccine performance by improving lymph-node localization and DC uptake [114], [115].

Additionally, cell-free aAPCs, engineered using polymeric scaffold coated with synthetic membranes displaying MHC-I/peptide complexes and co-stimulatory ligands, have shown superior activation of antigen-specific CD8^+^ cytotoxic T cells compared to natural DCs both in vitro and in vivo [103], [116]. Together, these platforms expand the versatility and translational potential of biomimetic nanovaccines.

Is thus the right place for the new figure 2 as you suggested to place it at thew end of section 3.

#### Advantages and remaining challenges

Artificial membranes provide GMP-compatible scalability, batch consistency, and lower risk of unintended immunogenicity due to the absence of endogenous proteins or nucleic acids [117], [118]. Moreover, their tunable physicochemical properties including membrane charge, fluidity, and drug release kinetics, support precise control over pharmacokinetics and immune activation profiles [46], [117].

Despite these advantages, key translational challenges remain. Major concerns including long-term biosafety of synthetic polymers, lipids, antigen density optimization, and risk of immune tolerance arising from suboptimal antigen spacing or orientation on the membrane surface [119], [120], [121], [122]. Addressing these issues through rigorous toxicological studies, rational antigen engineering, and standardized manufacturing will be essential to advance artificial membrane-based nanovaccines into late-stage clinical development.

To provide a consolidated visual overview of the innate and adaptive signaling cascades.

engaged by biomimetic nanovaccines, we summarize the major pathways involved in Fig. 2.

**Fig. 2:**
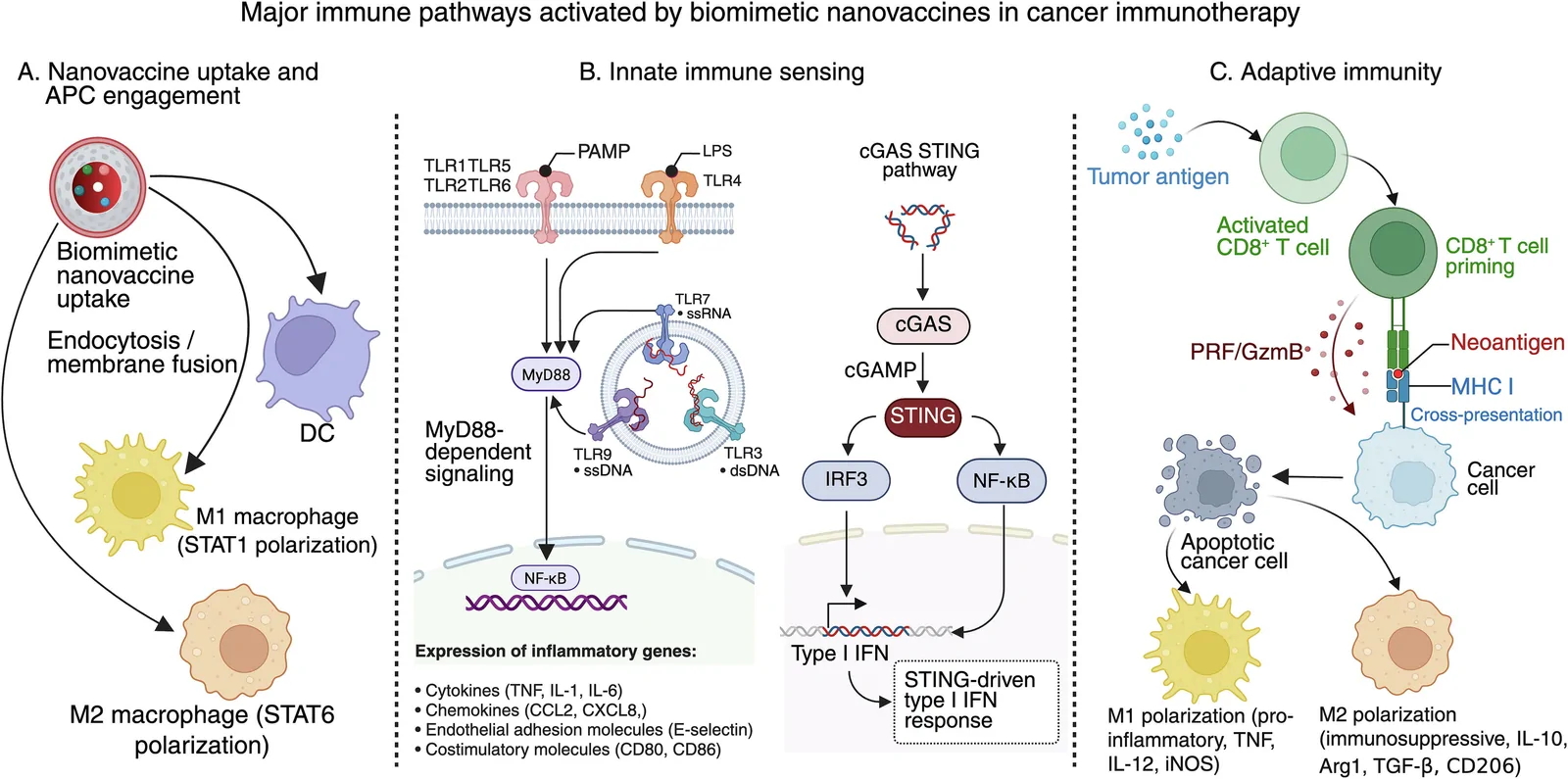
**Major immune pathways activated by biomimetic nanovaccines in cancer immunotherapy.** Biomimetic nanovaccines engage multiple innate and adaptive immune pathways to enhance anti-tumor responses. Following uptake by dendritic cells and macrophages, nanovaccines activate endosomal TLR7/8-MyD88-NF-κB/IRF7 signaling, inducing proinflammatory cytokines and type I interferons. Cytosolic delivery of nucleic acids or STING agonists triggers the cGAS-STING-TBK1-IRF3 pathway, further amplifying type I IFN production. Internalized antigens undergo proteasomal degradation and are transported via TAP for MHC-I cross-presentation, enabling efficient CD8^+^ T-cell priming. Concurrently, antigen presentation via MHC-II supports CD4^+^ T-cell activation. Nanovaccine components also modulate macrophage polarization, promoting either STAT1-driven M1 (proinflammatory) or STAT6-driven M2 (immunosuppressive) phenotypes depending on context. Together, these coordinated pathways support robust innate activation, cross-priming, and adaptive immunity critical for effective cancer immunotherapy.

## Core vaccine payloads: mRNA and peptide antigens

### Peptide-based nanovaccines

Peptide-based vaccines represent a cornerstone of cancer immunotherapy due to their high specificity, safety profile, and adaptability to personalized medicine. These platforms aim to elicit antigen-specific CTL responses by delivering defined tumor-associated or tumor-specific epitopes, typically derived from mutated or overexpressed proteins in cancer cells [16], [123]. However, free peptides are often poorly immunogenic, rapidly degraded, and inefficiently presented by APCs, limiting their clinical impact. Nanoparticle-based delivery systems have therefore emerged as powerful tools to improve peptide stability, lymphoid trafficking, and cross-presentation efficacy [20], [56].

#### Role in eliciting antigen-specific CTL responses

Peptide nanovaccines function by encapsulating or surface-loading antigenic peptides into nanoscale carriers that enhance their uptake by professional APCs, especially DCs [16]. Following internalization, nanoparticles release peptides into the endosomal or cytosolic compartments, facilitating processing and MHC class I (MHC-I) presentation [21]. This process drives the priming and activation of CD8^+^ T cells, which are crucial for effective tumor cell killing [18], [19], [20]. Co-delivery of TLR agonists or combination with ICIs can further enhance T cell expansion, improve functional avidity, and support long-term immune memory formation [104], [124].

#### Design and selection of tumor epitopes

The efficacy of peptide-based nanovaccines depends critically on the optimal selection and validation of tumor-specific epitopes. Key considerations include MHC-binding affinity, immunogenicity, and tumor specificity. Selected peptides must stably bind to MHC-I molecules while avoiding central tolerance and minimizing autoimmunity risk [9], [15], [17]. Neoantigens or overexpressed TAAs with minimal expression in normal tissues are typically prioritized.

Engineering heteroclitic peptides to enhance MHC binding or TCR engagement has also shown promise in boosting immune potency [125]. Advances in computational prediction, mass spectrometry, and next-generation sequencing have significantly accelerated epitope discovery, enabling more personalized vaccine design [126], [127].

Building on these technologies, our group has developed multiple nanovaccine platforms that target clinically relevant antigens with demonstrated translational potential.

#### Examples from our prior work: Survivin, BCMA, and STEAP peptides

Our group has contributed extensively to the development of peptide-loaded nanovaccines targeting high-value tumor antigens. We identified and optimized survivin (SV95) epitope variants using MHC stability assays and demonstrating that PLGA nanoparticle delivery enhanced cross-presentation and induced potent CTL responses in vitro [17]. In a separate study, BCMA (72–80) peptide-loaded nanoparticles, formulated using PLGA and liposomal carriers, significantly improved antigen delivery to human DCs and increased BCMA-specific CD8^+^ T cell activation, supporting their utility in multiple myeloma models [18]. Additionally, our work showed that six transmembrane epithelial antigen of the prostate (STEAP) peptides delivered via PLGA nanoparticles elicited robust CD8^+^ T cell responses and inhibited tumor growth in vivo, demonstrating the potential in prostate and other solid tumors [16].

Collectively, these studies underscore the translational feasibility of combining well-characterized peptide epitopes with biodegradable nanoparticle carriers to enhance CTL priming, expand antigen-specific immune responses, and drive therapeutic tumor regression.

### mRNA nanovaccines

Messenger RNA (mRNA) nanovaccines have emerged as a transformative immunotherapy modality owing to their versatility, safety, and capacity to induce potent cellular immune responses [128]. Unlike peptide-based vaccines, mRNA constructs can encode multiple epitopes or full-length antigens, enabling simultaneous activation of both CD8^+^ and CD4^+^ T cells following endogenous antigen expression within APCs [1], [104]. Formulated mRNA into nanoparticles protect it from degradation, promotes cytosolic delivery, and facilitates trafficking to lymphoid tissues where immune priming occurs [129].

#### Advantages: Rapid production and multi-antigen potential

mRNA nanovaccines offer several unique advantages. Their rapid, cell-free synthesis enables efficient incorporation of tumor neoantigens or shared TAAs, facilitating personalized vaccines manufacturing within clinically relevant timelines [10], [128], [130]. mRNA sequences selected through the same computational and immunopeptidomics pipelines used for peptide vaccines can encode multiple tumor-specific epitopes or full-length antigens, broadening antigenic coverage and reducing immune escape [131], [132].

Importantly, unmodified or partially modified mRNA exhibit intrinsic adjuvanticity through activation of innate immune sensors such as TLR3, TLR7/8, and RIG-I [133], [134]. This innate activation promotes DC maturation and enhances CTL priming. Because mRNA does not integrate into the genome and has predictable pharmacokinetics, it presents safety advantage over DNA or viral platforms [3].

#### Challenges: Instability and endosomal escape

Despite their therapeutic promise, mRNA nanovaccines face several critical biological and formulation challenges. Naked mRNA is rapidly degraded and thus requires encapsulation within protective carriers such as LNPs and polymeric nanoparticles [135], [136]. Even after successful cellular entry, poor endosomal escape remains a major bottleneck, as a significant portion of internalized mRNA undergoes lysosomal degradation rather than cytosolic release [137].

Dose optimization is another critical issue. Excessive mRNA or innate-stimulatory lipids may induce excessive inflammation, reduce antigen expression, or cause systemic toxicity [3], [138], [139]. Furthermore, the immunosuppressive TME can impair DC maturation and dampen antigen presentation, limiting the downstream activation tumor-specific T cells [13], [140], [141]. Therefore, addressing these barriers is crucial to improve the consistency, potency, and clinical reliability of mRNA-based cancer vaccines.

#### Dose-dependent reactogenicity and translational lessons from COVID-19 LNP vaccines

The global deployment of COVID-19 mRNA-LNP vaccines has provided unprecedented real-world insights into the dose–response behavior, immunogenicity, and safety of lipid nanoparticle platforms information directly relevant to cancer nanovaccine development [142]. A key observation from these vaccines is the dose-dependent nature of reactogenicity, driven by innate immune recognition of ionizable lipids and mRNA structures [133], [143]. At higher doses, early inflammatory responses such as fever, injection-site swelling, and transient lymphadenopathy are attributed to activation of pattern-recognition pathways including TLR7/8, RIG-I, and type I interferon signaling [65], [144].

Although innate activation is essential for initiating adaptive immunity, excessive stimulation can suppress antigen expression, impair tolerability, and limit dose escalation, which is a challenge especially relevant in oncology settings [145], [146]. Clinical and preclinical studies further demonstrate that optimal immune priming does not correlate with maximal inflammation; instead, doses that modulate innate activation while sustaining antigen expression achieve more favorable immune outcomes [145], [147].

A second translational insight concerns the chemistry of ionizable lipids, which strongly influences endosomal escape, biodistribution, and cytokine induction. Even subtle structural differences in ionizable lipids can markedly alter LNP tolerability and potency, underscoring the need for rational lipid engineering tailored for cancer vaccination [148], [149].

Finally, differences between healthy COVID-19 vaccine recipients and cancer patients who often exhibit immunosuppression, systemic inflammation, or altered pharmacokinetics highlight the importance of individualized dose optimization, careful immune monitoring, and iterative schedule refinement. These patient-specific considerations are essential to maximize antigen expression while avoiding excessive innate activation [3], [150].

Overall, the lessons learned from COVID-19 mRNA-LNP deployment provide a valuable framework for designing next-generation cancer nanovaccines, emphasizing controlled innate activation, dose-responsive formulation tuning, and strategic lipid engineering to harmonize immunogenic potency with real-world clinical tolerability.

Together, these insights provide a valuable framework for designing next-generation cancer mRNA nanovaccines that balance controlled innate activation with clinical tolerability through refined dosing, lipid engineering, and schedule optimization.

#### Innovations in mRNA stabilization and encapsulation

To address these challenges, several innovative strategies have been developed to enhance mRNA stability, delivery efficiency, and immunogenic performance. LNPs, such as those used in COVID-19 vaccines, remain the gold standard for mRNA delivery due to their ability to protect mRNA, facilitate endosomal escape, and minimize toxicity at physiological pH [135], [151], [152], [153]. Ligand functionalization like mannose, antibodies, or peptides can further improve APC targeting and lymph node homing [154].

Biodegradable polymeric carriers such as PLGA and PEI offer alternative encapsulation strategies with tunable release properties and can be engineered to co-deliver adjuvants or immune modulators [130], [155]. Incorporation of modified nucleosides (e.g., pseudouridine or N1-methyl-pseudouridine) enhances mRNA transcript stability, reduces innate immune sensing, and improves translational efficiency [156]. Hybrid nanoparticles combining lipid-polymer architectures or biomimetic coatings such as RBC membranes or exosomes provide additional improvements in pharmacokinetics, biocompatibility, and immune targeting [46], [94], [112].

Another promising approach is the use of self-amplifying mRNA (saRNA), which includes replicase elements enabling intracellular amplification of the antigenic payload, thereby reducing the required doses while prolonging antigen expression [157]. Collectively, these innovations are expanding the capabilities of mRNA nanovaccines, with emerging preclinical and early clinical evidence supporting their integration with ICIs and advanced delivery platforms [158]. Lessons from COVID-19 LNP vaccine dose-dependent reactogenicity relationships further emphasize the importance of optimizing dose, interval, and ionizable-lipid composition for oncology applications [159].

## Personalized and autologous nanoparticles

Personalized and autologous nanovaccines represent an advanced direction in cancer immunotherapy, enabling precise alignment between delivered immunogens and an individual patient’s tumor profile. By incorporating neoantigens, tumor lysates, or self-derived biomaterials including membranes from RBCs, leukocytes, tumor cells, or autologous exosomes, these platforms leverage biomimetic surface architecture to enhance immunogenicity, minimize off-target toxicity, and overcome tumor immune evasion [15], [95], [160]. Unlike conventional synthetic carriers, these platforms achieve personalization not only through antigen content, but also through biologically matched surface cues that influence trafficking, uptake, and immune activation.

### Autologous nanoparticle platforms and biomimetic coatings

Autologous nanoparticles fabricated from patient-derived materials (RBCs, leukocytes, tumor cell membranes, or exosomes) offer enhanced biocompatibility and enable biomimetic immune evasion and targeted delivery. These coatings offer several advantages: RBC- and leukocyte-derived membranes express CD47, conferring “self-recognition” and prolongs circulation via immune invisibility; tumor membrane coatings support homotypic binding to the originating tumor, enhancing tumor tropism and intratumoral accumulation [109], [161]; tumor-derived DAMPs or heat-shock proteins (HSPs) enhances innate immune activation and efficient DC activation and T-cell priming, strengthening downstream T-cell responses.

These biomimetic features significantly improve lymph node trafficking and enhance cross-presentation, positioning autologous coatings as highly efficient interfaces between nanocarriers and the host immune system. A comparative overview of RBC-, exosome-, and artificial- membrane coatings is provided in Table 1.

**Table 1:** Comparative features of major biomimetic coating strategies for cancer nanovaccines.

Coating type	Key biomolecular features	Immunological function	Advantages in vaccine delivery	Limitations / challenges	Representative applications	References
RBC membrane	CD47-SIRPα “don’t eat me” signal; phospholipid bilayer	Immune evasion; prolonged circulation	Long half-life, reduced clearance, stable antigen display	Lacks tumor tropism; limited innate stimulation	RBC-coated PLGA nanovaccines for prolonged antigen exposure	[43], [257]
Exosome membrane	Native tetraspanins (CD9, CD63), tumor-derived proteins	Efficient cell–cell communication; natural APC uptake	Intrinsic tumor targeting; supports cross-presentation; high biocompatibility	Batch variability; isolation complexity	Exosome-coated RNA or peptide nanovaccines enhancing DC activation	[258], [259]
Tumor cell membrane	Neoantigens, TAAs, DAMPs, HSP70/90	Homotypic targeting; strong immunogenicity	Personalized antigen repertoire; potent DC activation	Limited scalability; safety concerns (residual nucleic acids)	Tumor-lysate membrane vaccines and personalized nanovaccines	[15], [260]
Immune cell membrane (Macrophage, DC)	Chemokine receptors, phagocytic markers	TAM/TME interaction; myeloid niche engagement	Tumor homing; enhanced antigen transfer to APCs	Heterogeneous phenotype; regulatory hurdles	Macrophage-coated NPs for reprogramming TAMs to M1 phenotype	[261], [262]
Platelet membrane	Adhesion proteins (P-selectin)	Binds circulating tumor cells; vascular targeting	Supports metastasis-targeting strategies	Limited antigen content; thrombosis risk	Platelet-coated NPs for CTC targeting	[263], [264]
Artificial synthetic membrane	Tunable lipids, polymers, controlled antigen spacing	Highly customizable immune activation	GMP scalability; reproducible composition; adjustable antigen orientation	Lower biological complexity; potential long-term toxicity	Synthetic membrane nanovaccines with optimized antigen density	[46], [175]

Reviewer 4′s comment: Table 1, Table 2 are too short and do not provide informative knowledge for the readers. Please help how to expand Table 1, Table 2.

**Table 2:** Representative peptide-based and mRNA-based nanovaccine platforms and their preclinical/clinical performance.

Payload type	Antigen example	Delivery platform	Preclinical & clinical stage	Mechanistic immunological effect	Reported outcome	References
Peptide	Survivin (SV95)	PLGA-NP	Preclinical	Enhanced MHC-I cross-presentation	Potent CTL activation; tumor suppression	[17]
Peptide	BCMA (72–80)	PLGA + liposome	Preclinical	Improved DC uptake and peptide persistence	Strong CD8^+^ T-cell priming	[18]
Peptide	STEAP1	PLGA-NP	Preclinical	Metalloprotein antigen presentation	Tumor growth inhibition	[16]
Peptide (neoantigen Panel)	Patient-specific neoepitopes	Lipid-polymer hybrid	Preclinical	Polyclonal TCR expansion	Enhanced tumor rejection	[10]
mRNA (Personalized)	Neoantigen mix (individualized)	LNP	Phase I/II (e.g., mRNA-4157)	Endogenous antigen synthesis; CD8^+^ priming	T-cell expansion and ICB synergy	[265]
mRNA (shared antigen)	KRAS-G12D, WT1, gp100	Ionizable-LNP	Preclinical/early clinical	Robust type I IFN response	Potent tumor-specific immunity	[15]
mRNA (full-length tumor antigen)	NY-ESO-1, MUC1	Lipid-polymer hybrid NP	Preclinical	Broad CD4/CD8 epitope coverage	Overcomes antigen heterogeneity	[266]
Self-amplifying mRNA (saRNA)	Viral or tumor antigens	LNP or hybrid NP	Preclinical	Intracellular RNA amplification	Strong immune response at low dose	[267]
mRNA with biomimetic coating	Neoantigen panel	Exosome- or RBC-coated NP	Preclinical	Increased lymph-node trafficking	Higher CD8^+^ T-cell activation vs. uncoated LNP	[268]

### Peptide-based personalized nanovaccines

Peptide-based nanovaccines encapsulate patient-specific neoepitopes or shared TAAs to stimulate robust CTL responses [20], [104]. When delivered through biomimetic carriers such as PLGA nanoparticles with membrane coatings or liposome systems, these peptides gain enhanced serum stability, improved lymph node targeting, and efficient uptake by professional APCs such as DCs [17], [162].

Studies using survivin, BCMA, and STEAP peptides in nanoparticle systems have demonstrated potent antigen-specific immune activation and tumor control across multiple models [16], [17], [18]. Moreover, co-delivery of peptide antigens with immunological adjuvants or immune modulators further improve cross-priming while mitigating the risk of immune tolerance [1], [163].

Representative peptide- and mRNA-based nanovaccine platforms are summarized in Table 2.

### mRNA-based personalized nanovaccines

mRNA nanovaccines encoding patient-specific neoantigens or full-length tumor antigens and leverage intracellular translation to drive endogenous antigen synthesis in APCs [104], [164]. Delivery via biomimetic carriers including LNPs, hybrid lipid-polymer nanoparticles, or membrane-coated systems confers enhanced endosomal escape, improved DC tropism, and stronger CD8^+^ T-cell priming [165].

Recent advances in next-generation sequencing and AI-guided epitope prediction have markedly accelerated the identification of patient-specific neoepitopes, enabling rapid and individualized mRNA vaccine design [10], [166]. When delivered through optimized biomimetic platforms such as LNPs and lipid-polymer hybrid nanoparticles, these mRNA constructs induce robust antigen-specific CD8^+^ T cell responses in both preclinical systems and early clinical trials [104], [167]. Notably, the mRNA-4157 trial further demonstrates the clinical feasibility and therapeutic synergy of combining personalized mRNA nanovaccines with immune checkpoint blockade (ICB), supporting their integration into next-generation precision immunotherapy regimens [9], [10], [168].

### Tumor lysate-based and exosome-coated vaccines

Tumor lysates offer a broad, individualized antigenic repertoire that can be encapsulated within or coated onto nanoparticle platforms for cross-presentation [169], [170]. When the lysate is used as the membrane coating itself, it preserves the full spectrum of patient-specific tumor antigens and enhances homotypic targeting [30], [171].

Autologous exosomes represent another powerful biomimetic system. Their endogenous surface markers and RNA cargo enable physiologic intercellular immune signaling and natural DC uptake pathways [80], [172]. Engineered exosomes used as nanovaccine carriers or as synthetic aAPCs expanding design flexibility and permit modulation of immune signaling [25], [173].

### Translational and manufacturing challenges

Despite their strong therapeutic promise, personalized biomimetic nanovaccines face substantial translational challenges [31], [174]. A primary barrier is the sourcing and consistency of patient-derived materials, which require stringent sterile processing and inherently exhibit heterogeneity [175], [176], [177]. This variability can compromise the reproducibility and reduce the overall efficacy. Additionally, scalability and manufacturing turnaround remain major concerns, particularly for patients with rapidly progressing cancers who require timely treatment [178]. Current fabrication workflows often fail to meet the necessary speed for clinical deployment. Regulatory classification further complicates translation, as these platforms merge the characteristics of biologics, tissue-derived materials, and medical devices, leading to ambiguity in approval pathways [179].

Cost-effectiveness presents another critical issue, underscoring the need for automated, modular, and GMP-compatible manufacturing pipelines to support broader clinical and large-scale applications [158]. Addressing these interconnected challenges is essential for advancing personalized biomimetic nanovaccines from early-stage studies into routine precision oncology.

The development of biomimetic nanoplatforms for cancer immunotherapy requires deliberate attention to several core design parameters that collectively determine clinical performance, manufacturability, and regulatory readiness. A central consideration is antigen selection and validation. Neoantigens, TAAs, or lysate-derived antigen pools must exhibit sufficient immunogenicity while minimizing the risk of autoimmune activation, and integration of NGS-guided epitope prediction with mass spectrometry-based antigen validation improves specificity and personalization [10], [166].

Equally important is the stability and structural integrity of the biomimetic coating. Membrane-derived shells whether sourced from RBCs, exosomes, tumor cells, or CAFs must preserve native protein orientation, membrane fluidity, and compositional fidelity during storage, transport, and circulation. Robust batch-to-batch quality control, including assays that assess coating density and vesicle fusion consistency, is essential for reproducible manufacturing [109], [161].

Immune targeting properties also play a defining role in platform performance. Biomimetic surfaces enriched with markers such as CD47, chemokine receptors, integrins, or tumor-specific adhesion molecules can fine-tune interactions with APCs, promote lymph-node trafficking, and enhance cross-presentation efficiency [28], [57], [81]. These elements directly shape biodistribution, immune-cell engagement, and downstream T-cell priming.

From a translational perspective, scalable and GMP-compatible manufacturing remains a critical requirement. Standardized membrane isolation, purification, and nanoparticle assembly workflows supported by automated extraction technologies, microfluidic formulation methods, and sealed sterile systems are essential to achieve regulatory-grade consistency [175], [176]. Ensuring endotoxin control, sterility, and validated release criteria further strengthens the regulatory pathway.

Finally, safety considerations must account for both the immunogenicity balance and degradation profiles of the biomimetic components. Platforms should demonstrate predictable biodegradation kinetics, absence of residual donor nucleic acids when applicable, and minimal off-target immune activation. Comprehensive toxicological assessment is particularly important for hybrid systems that incorporate synthetic linkers or engineered lipid-polymer interfaces [119], [120], [122].

Collectively, these design criteria provide a structured foundation for advancing biomimetic nanovaccines toward clinical translation. By integrating principled antigen selection, stable membrane engineering, immune-targeting precision, GMP-ready manufacturing processes, and rigorous safety evaluation, biomimetic platforms can achieve the potency, reproducibility, and regulatory clarity required for personalized cancer immunotherapy.

Fig. 3 presents an overview of the workflow from tumor biopsy to personalized nanovaccine formulation and clinical evaluation. Fig. 4 integrates schematics and a comparative summary of representitive nanovaccine platforms, antigen types, delivery routes, and clinical phase across cancer types.

**Fig. 3:**
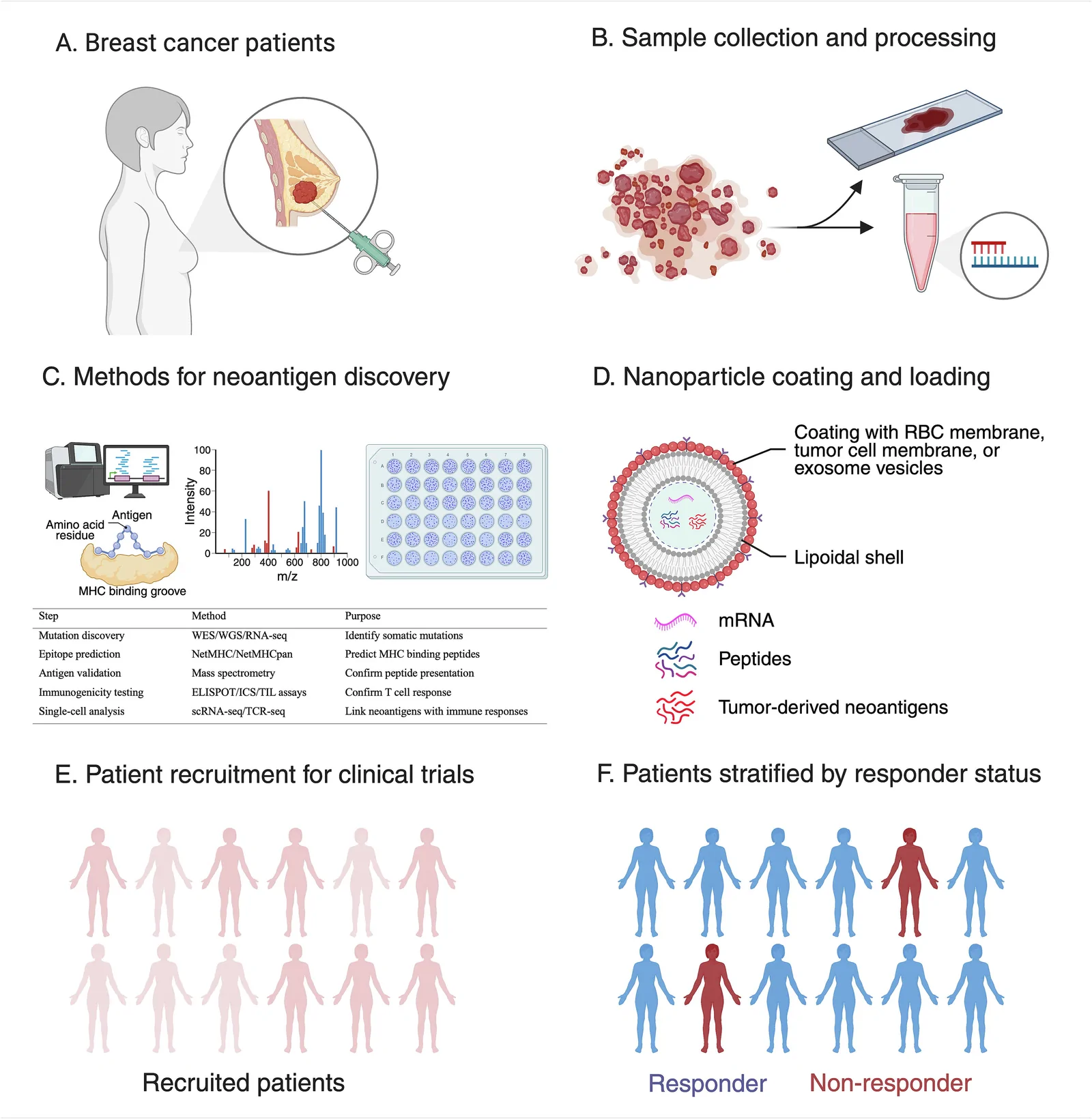
Personalized nanovaccine design workflow for cancer immunotherapy. This schematic illustrates the key steps involved in the development and clinical translation of personalized nanovaccines. (A) Breast cancer patients undergo tumor biopsy to obtain patient-specific tumor material. (B) Collected tumor samples are processed for downstream molecular and pathological analyses. (C) Neoantigen discovery is conducted using genomic sequencing, bioinformatic prediction of MHC-binding epitopes, and immunogenicity validation assays. (D) Identified mRNA, peptides, or tumor-derived neoantigens are incorporated into nanoparticle platforms via coating with RBC membranes, tumor cell membranes, or exosome vesicles. (E) Eligible patients are recruited into clinical trials receiving the personalized nanovaccine formulations. (F) Post-treatment, patients are stratified based on therapeutic response (responder vs. non-responder) for outcome evaluation and immune monitoring. This workflow integrates precision oncology, nanotechnology, and immunotherapy to support individualized cancer treatment strategies.

**Fig. 4:**
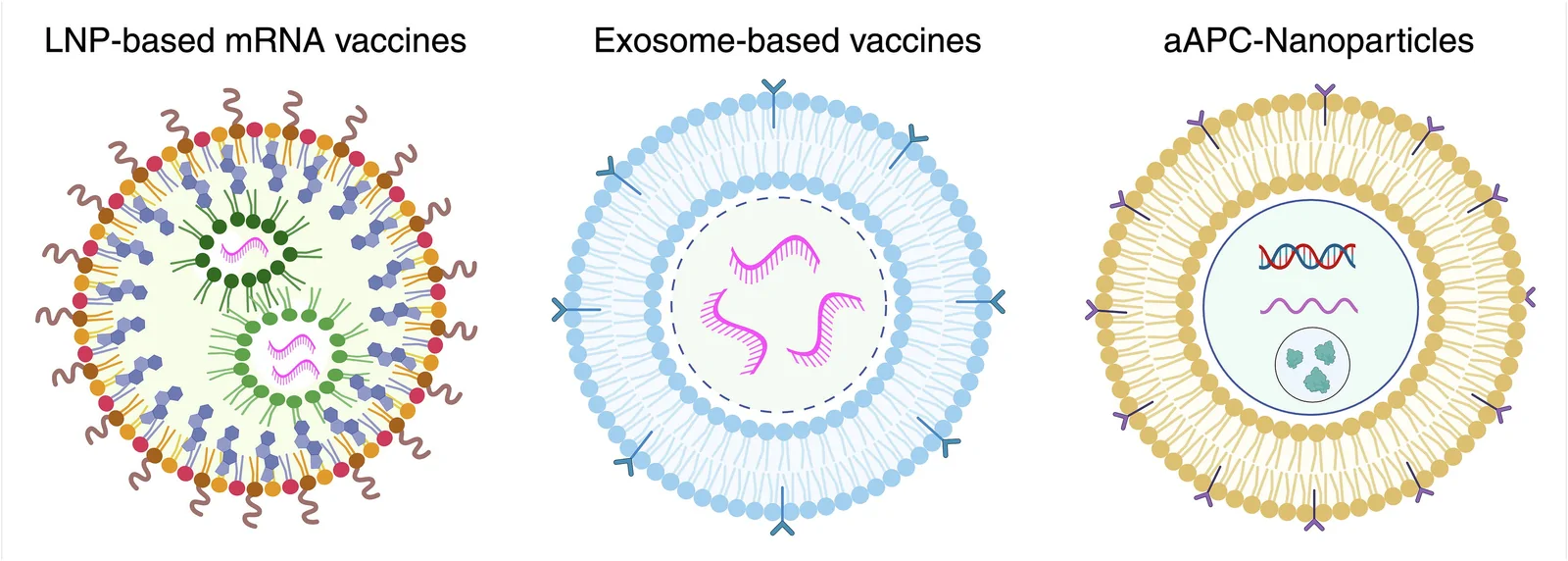
**Ongoing clinical trials of personalized and biomimetic nanovaccines.** Representative platforms include LNP-based mRNA vaccines (e.g., BNT111, mRNA-4157) demonstrating induction of both de novo and boosted pre-existing T-cell responses in melanoma, exosome-based vaccines paired with ICB, and aAPC-mimicking nanoparticles co-loaded with tumor antigens and adjuvants. These clinical efforts collectively highlight the feasibility and translational potential of biomimetic and personalized nanovaccine platforms in cancer immunotherapy.

Given the complexity of personalized vaccine development, Table 3 summarizes the major and methodologies involved in neoantigen discovery from mutation identification to validation of immunogenicity and T cell reactivity.

**Table 3:** The main methods for neoantigen discovery.

Step	Method	Purpose	References
Mutation discovery	WES/WGS/RNA-seq	Identify somatic mutations	[269]
Epitope prediction	NetMHC/NetMHCpan	Predict MHC binding peptides	[270]
Antigen validation	Mass spectrometry	Confirm peptide presentation	[271]
Immunogenicity testing	ELISPOT/ICS/TIL assays	Confirm T cell response	[272]
Single-cell analysis	scRNA-seq/TCR-seq	Link neoantigens with immune responses	[273]

## Combination strategies with nanovaccines

Biomimetic nanovaccines offer a versatile platform for combination therapy by integrating antigen delivery with immune modulation, lymph-node targeting, and enhanced cross-presentation. Their membrane coatings derived from RBCs, tumor cells, macrophages, CAFs, or exosomes fundamentally reshape biodistribution and immune-cell engagement, enabling stronger synergy with immune checkpoint blockade (ICB), innate immune agonists, macrophage-reprogramming strategies, and radiotherapy [104], [180]. These combinations collectively transform “cold” tumors into inflamed, T-cell-infiltrated lesions capable of responding to immunotherapy.

### Immune checkpoint blockade (ICB)

ICB is most effective when tumors contain primed, antigen-specific T cells capable of responding to PD-1, PD-L1, or CTLA-4 inhibition [181]. Many solid tumors lack this pre-existing immunity, leading to poor responses with ICB monotherapy. Biomimetic nanovaccines overcome this limitation by enhancing antigen trafficking to lymph nodes, enhancing DC uptake, and inducing de novo CD8^+^ T-cell priming through controlled antigen presentation [182], [183].

When nanovaccines precede ICB, the newly primed T cells persist longer and exhibit stronger reinvigoration within the immunosuppressive TME [183], [184]. Moreover, biomimetic carriers such as RBC membranes or exosome-coated nanoparticles further enhance delivery precision, prolong circulation, and minimize systemic toxicity, which is particularly valuable when combined with ICB [38], [40].

Optimal scheduling is crucial, priming with nanovaccines before ICB consistently outperforms simultaneous delivery [14], [185]. Low dose, repeated nanovaccine administration helps maintain antigen presentation and sustain T cell responses [186]. Emerging co-delivery strategies package both tumor antigens and ICB (anti-PD-1 peptides, PD-L1 siRNA) within a single nanoparticle, enabling synchronized activation at the tumor site and reducing the need for systemic antibody infusion [102], [187], [188]. These integrated systems highlight how biomimetic coatings can control spatial and temporal coordination to enhance ICB efficacy.

### TLR agonists, STING agonists, and adjuvants

Innate immune agonists, especially TLR ligands and STING activators, serve as potent adjuvants that amplify nanovaccine performance. Biomimetic nanocarriers enable co-delivery of antigen and adjuvant within the same vesicle, ensuring synchronized intracellular uptake and activation of DCs [65], [124]. Encapsulated TLR7/8 (R848), TLR9 ligands (CpG), or STING agonists (cGAMP) induce robust type I interferon signaling, pro-inflammatory cytokine production, and DC maturation, all of which improve antigen cross-presentation and CD8^+^ T cell priming [189]. This activation promote the migration of activated APCs to lymph nodes and induces chemokines such as CXCL9/10 that recruits effector T cells to the tumor site [146], [190], [191].

Biomimetic systems such as exosome-coated nanocarriers and aAPC-mimicking formulations enable coordinated delivery of antigen-adjuvant combinations and have outperformed conventional nanoparticles in multiple preclinical models [9], [95].

To contextualize coating strategies, Table 4 summarizes compares the immunological and translational properties of RBC membranes, exosomes, and synthetic/artificial coatings.

**Table 4:** Comparison of coating strategies (RBC vs. exosome vs. artificial).

Coating Type	Source	Immune Evasion	Targeting Capability	Payload Protection	Scalability	Clinical Translation Potential	References
RBC Membrane	Autologous or allogeneic RBCs	High (CD47-mediated)	Low (passive)	Strong	High	High	[274]
Exosome	Tumor/immune/stem cells	Moderate (variable)	High (homing properties)	Moderate	Moderate to low	Emerging	[81]
Artificial Membrane	Synthetic lipids/polymers	Low (depends on design)	High (customizable)	Strong	High	Moderate to high	[275]

Beyond RBC and exosome coating systems, immune-cell-derived membranes add further specialization. Macrophage membranes can confer tumor-homing and cytokine-interfacing properties, platelet membranes support adhesion to damaged vasculature or circulating tumor cells, and leukocyte membranes facilitate immune evasion and homing to inflamed-tissue [110], [192]. These advanced coatings deepen biological integration but introduce additional heterogeneity and QC complexity during scale-up.

### *Macrophage-targeted and* TAMs*-reprogramming strategies*

Given the central role of tumor-associated macrophages (TAMs) in suppressing antitumor immunity [193], [194], biomimetic nanovaccines increasingly incorporate macrophage-directed design. Ligand-decorated nanocarriers bearing mannose, scavenger-receptor and folate ligands selectively target macrophages and DCs while facilitating antigen uptake and processing. When combined with TLR or STING agonists (e.g., MPLA, CpG, cGAMP), these formulations promote repolarization of immunosuppressive M2-like macrophage (M2) into a pro-inflammatory M1-like (M1) phenotype, enhancing antigen cross-presentation and enhancing downstream T-cell infiltration [195].

Additional approaches integrate TAM-targeted inhibitors such as CSF1R or PI3Kγ inhibition, which deplete or reprogram suppressive TAMs to support a more inflamed TME [196]. By simultaneously directing antigens to lymph-node and remodeling the myeloid niche, biomimetic nanovaccines achieve synergistic amplification of vaccine-induced T-cell responses.

### Radiotherapy and other modalities

Beyond immunomodulatory agents, physical therapies especially radiotherapy provide a powerful complement to biomimetic nanovaccines by reshaping the TME and releasing tumor antigens [197], [198]. Radiation induces immunogenic cell death (ICD) [197]. producing TAAs and damage-associated molecular patterns (DAMPs) such as calreticulin and high mobility group box 1 (HMGB1), which recruit and activate DCs [199]. It also increases MHC class I expression and activates the cGAS-STING pathway, thereby enhancing type I interferon signaling [200], [201].

When administered together, nanovaccines amplify these radiation-induced signals by delivering antigens, including those liberated by irradiation to DCs, enabling stronger and more durable CD8^+^ T-cell responses [25], [104], [198]. Radiotherapy also converts the tumor itself into an in-situ vaccine, and nanocarriers can be engineered to release cargo in response to radiation-induced ROS or pH changes, ensuring precise delivery to irradiated tissue [102], [202].

Other modalities such as high-intensity focused ultrasound (HIFU), photothermal therapy, and cryoablation similarly increase antigen availability and can be integrated with membrane-coated nanovaccines to promote epitope spreading and T-cell infiltration [203], [204]. Collectively, these multimodal approaches position biomimetic nanovaccines as foundational components of combination immunotherapy.

Beyond local effects, radiotherapy can also induce a systemic antitumor response known as the abscopal effect, in which tumor regression occurs at distant, non-irradiated lesions. This phenomenon arises when radiation-released TAAs and DAMPs drive robust DC activation, resulting in systemic expansion and trafficking of tumor-specific CD8^+^ T cells. Although historically rare, the abscopal effect becomes substantially more frequent when radiotherapy is paired with immunomodulatory therapies such as ICB or biomimetic nanovaccines. Nanovaccines enhance this process by improving antigen capture, promoting efficient cross-presentation, and sustaining systemic T-cell responses, thereby transforming localized irradiation into a body-wide immune activation capable of controlling metastatic disease.

## Clinical translation and regulatory considerations

The remarkable success of mRNA vaccines during the COVID-19 pandemic has accelerated efforts in adapting similar nanovaccine platforms for cancer immunotherapy [205]. However, translating biomimetic and personalized nanovaccines from bench to bedside remains multifaceted challenges that span immuno-oncology, nanotechnology, and regulatory science [31], [104].

### Current clinical trials

Most ongoing clinical trials continue to rely on conventional lipid nanoparticle (LNP) formulation, a platform validated by the large-scale rollout of mRNA vaccines and the associated technological advances established during the COVID-19 vaccine era [159], [206]. More recently, biomimetic and personalized nanovaccines have entered early-phase clinical evaluation, aiming to elicit durable, antigen-specific, and precisely targeted antitumor immunity [35], [207].

Among leading examples, BioNTech’s BNT111 (NCT02410733) targets four melanoma-associated TAAs: NY-ESO-1, MAGE-A3, Tyrosinase, and TPTE, demonstrating induction of both de novo and boosted pre-existing T-cell responses in advanced melanoma [208]. In the Phase II KEYNOTE-942 study, the combination of mRNA-4157 with pembrolizumab reduced the risk of recurrence by 44% and extended median progression-free survival (PFS) from 16 to 23 months in resected high-risk melanoma [209]. Additional BioNTech programs BNT112 (prostate cancer, NCT04382898), BNT113 (HPV-driven tumors, NCT04534205), BNT114 (breast cancer), BNT115 (ovarian cancer), and BNT116 (NSCLC, NCT05142189) are under active investigation [210].

Additionally, Moderna and Merck co-developed personalized mRNA vaccines mRNA-4157 and mRNA-5671, designed to deliver patient-specific neoantigens for tumors such as melanoma and NSCLC [211], [212]. These LNP-delivered platforms exhibit favorable safety and strong immunogenicity, confirming the clinical viability of individualized antigen design [213]. Other innovative strategies include exosome-based vaccines, such as Dex (DC-derived exosomes), which mimic natural antigen presentation mechanisms and aAPC-mimicking nanoparticles, engineered to directly prime cytotoxic T cells in situ [19], [113], [214].

Although most candidates remain in phase I-II trials, they collectively highlight a growing commitment to precision immunotherapy that integrates personalized neoantigen antigens and biomimetic delivery, and synergistic combination with ICIs or chemotherapy [123]. Despite encouraging feasibility and safety data, clinical efficacy remains variable. For instance, BNT111 induced tumor-specific T cell responses and disease stabilization in melanoma (NCT04526899), but objective response rate (ORR) remained modest due to interpatient variation in antigen presentation and immune history [210], [211]. Exosome-based vaccines, despite demonstrating immunogenicity, continue to face manufacturing and scalability barriers compared to DC-based approaches.

Fig. 4 integrates schematics and a comparative summary of representative nanovaccine platforms, antigen types, delivery routes, and clinical phases across cancer types. Table 5 summarizes key elements of select trials, including antigen type, delivery platform, cancer indication, administration route, and trial phase.

**Table 5:** Summary of clinical trials using personalized and biomimetic nanovaccine platforms.

Platform	Example Vaccine	Antigen Type	Combination Strategy	Trial Phase	Cancer Type	Delivery Route	References
LNP	BNT111 / mRNA-4157	Neoantigens	None / ICB	Phase II	Melanoma / NSCLC	Intramuscular	[208], [212]
Exosome	Exosome-based vaccine	Tumor antigens + ICB	ICB	Phase I	Melanoma / NSCLC	Intravenous	[89], [276]
Artificial APC	aAPC NP	Tumor antigens + adjuvant	Chemotherapy	Phase I	Breast / Colorectal	Intravenous / Intratumoral	[25], [103]

### Regulatory, safety, and translational challenges

The use of patient-derived materials including tumor lysates, RBC membranes, and exosomes adds considerable complexity to the regulatory landscape [111], [215]. These products often blur traditional boundaries between biologics, cell-based therapies, and combination products [216]. A major regulatory concern lies in GMP compliance, where per-patient variability, sterility validation, and classification ambiguities must each be addressed [217]. Small-batch personalized production increases QA/QC and cost burdens, while multi-agency oversight (e.g., biologic + drug divisions) further complicates approval for combination therapies such as nanovaccines with checkpoint inhibitors [218].

To provide an integrative view, Fig. 5 maps these regulatory and safety challenges of nanovaccine development to corresponding biomimetic mitigation strategies. Upstream manufacturing constraints such as GMP standardization, sterility testing, and product classification are linked to downstream safety concerns (e.g., immunotoxicity, off-target effects) and are mapped to targeted biomimetic solutions that support clinical scalability and reproducibility.

Building on the regulatory and safety framework summarized in Fig. 5, Table 6 compares leading nanovaccine platforms across key criteria, including antigen delivery strategies, immunogenicity, scalability, and regulatory complexity. As illustrated in Fig. 6, overcoming the scalability and QA/QC barriers will require a multifaceted approach combining advanced engineeing platforms with regulatory alignment to achieve widespread clinical adoption..

**Fig. 5:**
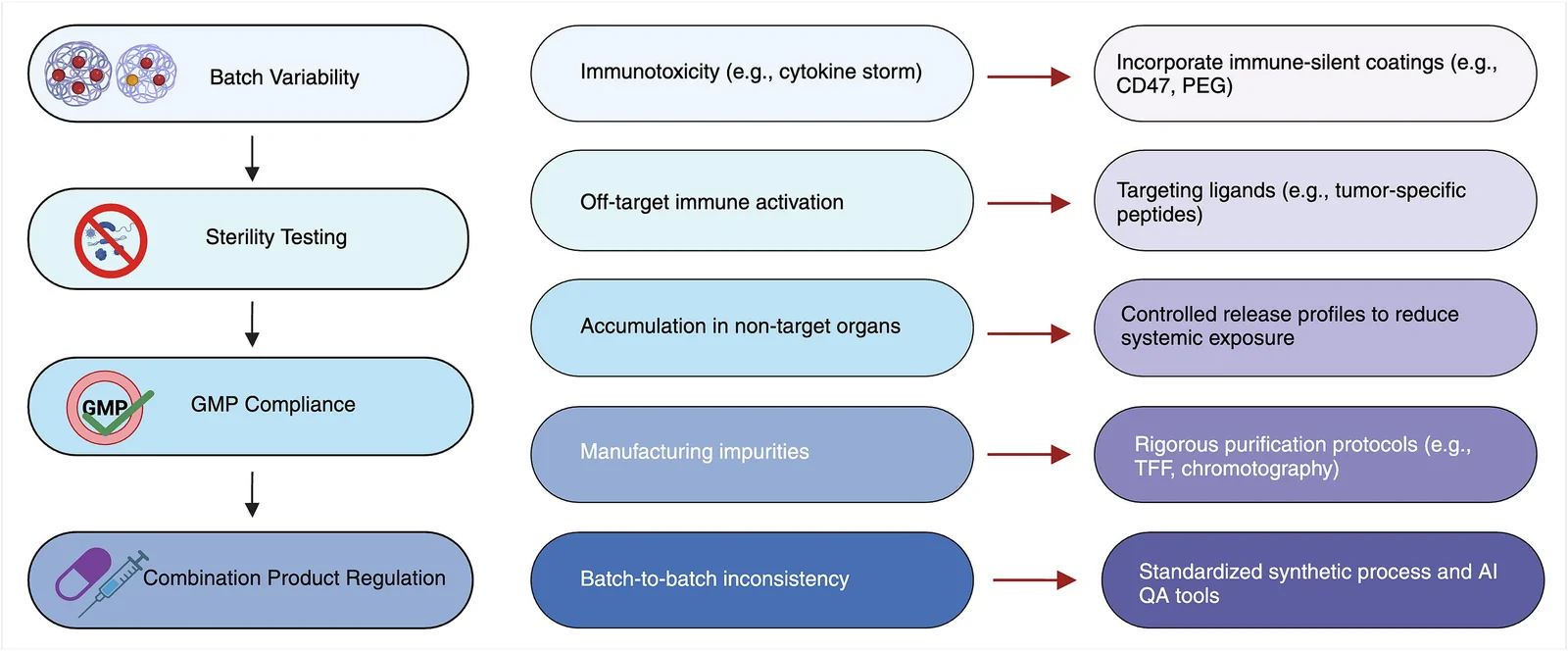
**Integrated barriers and mitigation strategies in personalized nanovaccine development.** This schematic presents a consolidated overview of key obstacles in the clinical translation of personalized nanovaccines, spanning GMP compliance, sterility testing, immunotoxicity, and off-target accumulation. The left column highlights regulatory and manufacturing constraints, including batch variability and combination product classification. The center column captures major safety concerns such as cytokine storms, non-target immune activation, and residual impurities. The right column illustrates corresponding biomimetic and engineering solutions ranging from immune-silent coatings and ligand-directed targeting to purification protocols and AI-guided QA tools aimed at enhancing safety, consistency, and clinical scalability.

**Table 6:** Comparative summary of key performance characteristics of nanovaccine platforms.

Feature / Criteria	LNP-Based mRNA Vaccines	Exosome-Based Vaccines	aAPC-Mimicking Nanoparticles	References
Antigen loading	mRNA encoding neoantigens	Endogenous/exogenous tumor proteins or peptides	Tumor antigens + adjuvants	[232]
Immune modulation	Primarily CD8^+^ T cells (via translated antigen)	Multimodal (CD4^+^, CD8^+^, innate immunity)	Direct activation of cytotoxic T cells	[104]
Delivery route	Intramuscular	Intravenous	Intravenous / Intratumoral	
Scalability	High (RNA synthesis & LNP formulation scalable)	Limited (patient-specific exosome isolation)	Moderate (batch synthesis with quality variation)	[277]
Stability & storage	Cold chain required	Medium stability	Variable; often requires cold storage	
Manufacturing complexity	Moderate (standardized RNA + lipid chemistry)	High (biological purification and QC complexity)	High (bioengineering + co-delivery formulation)	[278]
Immunogenicity consistency	Moderate-high (depends on antigen design)	Variable (donor-derived variability)	High (adjuvant-enhanced activation)	[279]
Clinical maturity	Phase II (e.g., BNT111, mRNA-4157)	Phase I (e.g., Dexosome)	Phase I (e.g., Dexosome)	[280]

**Fig. 6:**
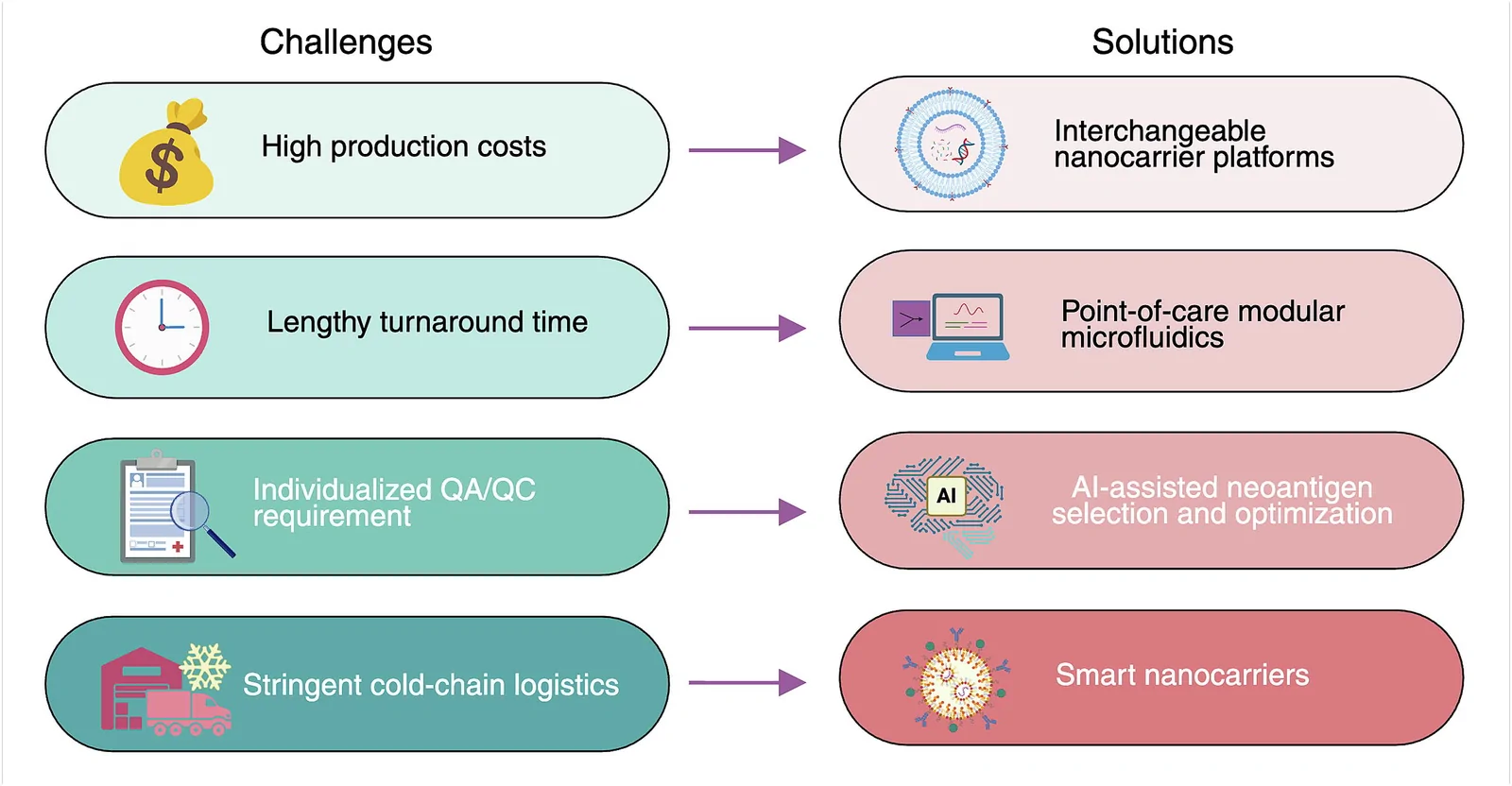
**Overcoming scalability challenges in personalized nanovaccine manufacturing.** Key barriers to clinical deployment include high production costs, prolonged turnaround time, individualized QA/QC requirements, and cold-chain storage demands. Proposed solutions include modular antigen-loading nanocarriers to reduce costs, decentralized manufacturing via point-of-care microfluidics, AI-driven neoantigen design to optimize efficiency, and smart nanocarriers to improve formulation stability. Together, these integrated strategies improve scalability, reduce production burden, and enhance clinical accessibility of personalized nanovaccines.

These distinctions emphasize that while LNP systems currently dominate clinical testing due to their scalability and platform familiarity, exosome- and aAPC-based platforms represent biologically sophisticated but technically demanding alternatives [219]. Strategic selection based on disease context, patient population, and regulatory feasibility will be critical to guide future development pathways.

### Scalability and quality considerations

Beyond regulatory compliance, achieving scalable and efficient production of personalized nanovaccines remains a critical hurdle. Each patient-specific batch requires individualized antigen sequencing, customized formulation, and comprehensive quality-assurance and control (QA/QC) testing under time-sensitive conditions, particularly for patients with rapidly progressing malignancies. Furthermore, many mRNA-based nanovaccine platforms rely on strict cold-chain logistics, further complicating distribution and long-term storage in resource-limited settings.

Emerging innovations including modular microfluidic manufacturing, AI- and LLM-assisted neoantigen design, and interchangeable nanocarrier platforms are being developed to streamline production and enhance adaptability [7], [220]. These integrated systems aim to bridge the gap between personalization and manufacturability, enabling rapid and flexible vaccine generation tailored to individual patients. Continued progress in automation, bioengineering, and regulatory harmonization will be essential to accelerate the clinical translation while maintaining batch consistency and safety.

As illustrated in figure 5, overcoming the scalability and QA/QC barriers will require a multifaceted approach combining advanced engineering platforms with regulatory alignment to achieve widespread clinical adoption.

### Safety considerations and engineering strategies

In addition to regulatory and scalability challenges, safety remains a major concern for the clinical adoption of personalized nanovaccines. Key risks include cytokine release syndrome, off-target immune activation, undesired biodistribution to non-target organs, and residual impurities from manufacturing impurities [14], [221]. These toxicities may arise from uncontrolled adjuvant release, excessive innate-immune stimulation, or incomplete purification of biological coatings.

As summarized in Fig. 5, biomimetic and engineering strategies provide multiple avenues to mitigate these risks. These approaches include immune-silent surface coatings (e.g., CD47, PEG) that reduce nonspecific phagocytosis and complement activation; ligand-directed targeting to tumor-associated receptors that confine immune activation to the tumor site, controlled-release formulations that synchronize antigen and adjuvant kinetics to prevent systemic cytokine surges; and rigorous purification and microfluidic quality-control pipelines to remove endotoxin nucleic-acid fragments, and protein contaminants that could otherwise trigger inflammatory or autoimmune responses [48], collectively enhancing the safety profile of personalized nanovaccines and enhancing regulatory confidence for clinical translation.

## Knowledge gaps and future directions

Despite significant advancements in biomimetic and personalized nanovaccine design, several translational barriers remain unresolved. These include incomplete understanding of immune interactions, insufficient predictive tools for antigen selection, and challenges in harmonizing manufacturing with clinical trial design. Addressing these limitations will be pivotal for realizing nanovaccines as clinically viable immunotherapies, particularly in synergy with immune checkpoint blockade (ICB) and next generation immunomodulators [159], [222].

### Immunological mechanisms of biomimetic coatings

Biomimetic coatings including RBC-, exosome-, immune-cell-derived, and synthetic membrane interfaces substantially enhance nanoparticle stability, circulation time, and biodistribution. However, their immunological consequences remain incompletely understood. A central mechanistic question is how these coatings influence APC engagement, particularly among cDC1, cDC2, and macrophage subsets, which differ markedly in their capacities for cross-presentation, cytokine secretion, and migration to draining lymph nodes [223], [224]. Coating composition including lipid arrangement, glycosylation patterns, protein orientation, and surface charge likely modulates how efficiently nanoparticles are recognized, internalized, and processed by these APC subsets. Preferential uptake by cDC1 may promote robust MHC-I cross-presentation and CD8^+^ T-cell priming, whereas macrophage-dominant uptake in certain microenvironments may favor antigen degradation or tolerogenic signaling [225], [226].

Despite these emerging insights, it remains unclear under which physiological or tumor-associated conditions exosome- or autologous membrane-coated nanoparticles elicit immunostimulatory versus immunoregulatory outcomes. Additional mechanistic variables including endosomal escape efficiency, MHC-I loading fidelity, and the contribution of membrane glycan signatures to pattern-recognition receptor (PRR) engagement are not yet well defined [227], [228]. These uncertainties underscore the need for systematic evaluation of how biomimetic interfaces shape cross-presentation dynamics, T-cell priming thresholds, and downstream immune activation.

Advanced analytical platforms including intravital microscopy, single-cell RNA sequencing (scRNA-seq), high-dimensional immune profiling, and live-cell imaging of antigen trafficking are increasingly used to resolve the mechanistic uncertainties surrounding biomimetic coatings [229], [230]. These technologies will be essential for defining how specific membrane features influence APC subset engagement, intracellular routing, endosomal escape, and MHC-I antigen loading. By enabling quantitative mapping of antigen processing pathways and T-cell priming dynamics in vivo, such approaches are expected to clarify how different coating strategies shape the magnitude, quality, and durability of antitumor immune responses. Strengthening this mechanistic understanding will facilitate the rational engineering of next-generation biomimetic nanovaccines optimized for precision cancer immunotherapy [231].

### Predictive biomarkers and AI-driven personalization

The therapeutic efficacy of personalized nanovaccines depends on the accurate selection of tumor antigens capable of inducing durable and functional T cell responses [104], [180], [232]. However, current neoantigen prediction algorithms remain limited in correlating predicted computational binding affinity with actual immunogenicity in vivo [17], [233]. This gap underscores the need for validated predictive biomarkers that can inform patient selection, optimize dosing strategies, and guide personalized vaccine design. Biomarkers such as T cell receptor (TCR) repertoire diversity, tumor mutational burden (TMB), and immune infiltration profiles are emerging as surrogate indicators of response to nanovaccine-based immunotherapy [234], [235]. Incorporating these parameters into clinical decision-making frameworks will improve trial efficiency, enhance response prediction, and accelerate regulatory approval.

Advances in AI and large language model (LLM)-assisted analytics are transforming this landscape. AI-driven pipelines trained on expanding immunopeptidomics**,** transcriptomic, and clinical datasets are increasingly capable of modeling process such as MHC binding, antigen processing, and TCR engagement [127], [236]. Simultaneously, the development of ML-based nanoparticle simulation allow optimization of physicochemical parameters such as biodistribution, release kinetics, and lymph-node trafficking to tailor delivery platforms for specific tumor and immune contexts [24], [30]. The integration of multi-omics data with AI-assisted design holds the potential to enable real-time, patient-specific vaccine integration, creating a tightly coupled workflow that bridges computational prediction with clinical translation. Coupling biosensor modules (e.g., pH/ROS/protease-responsive gates) with AI-tuned release profiles may ultimately support closed-loop, microenvironment-responsive antigen and adjuvant delivery in vivo [237], [238].

In parallel with these computational innovations, the emergence of “green biomimetic nanovaccines” introduces a complementary avenue for sustainable and scalable vaccine design. Green biomaterials including plant-derived nanovesicles, polysaccharide-based shells, and microalgae-derived biopolymers offer intrinsic biocompatibility, low immunogenicity, and environmentally sustainable sourcing compared with conventional synthetic or cell-derived coatings [239], [240]. These materials can be engineered to mimic membrane-like structures, facilitate lymph-node targeting, and support antigen and adjuvant co-loading, making them attractive candidates for next-generation biomimetic vaccine carriers [241]. Their natural abundance and low-cost production platforms also align with GMP and regulatory expectations for reproducibility and safety [242]. Nevertheless, challenges remain, including batch-to-batch variability, limited control of surface ligand composition, and the need for rigorous toxicological evaluation to ensure predictable in vivo degradation and immune compatibility. As green biomaterials continue to gain traction in cancer therapy, integrating them into the design of biomimetic nanovaccines may provide a sustainable and clinically accessible path forward, particularly when combined with AI-guided optimization and predictive biomarker frameworks [174].

### Clinical trial design considerations and AI-enabled optimization

Clinical evaluation of biomimetic or personalized nanovaccines, particularly in combination with ICB, remains limited, but rapidly evolving [4], [183]. Successful clinical translation requires trial frameworks that accommodate individualized manufacturing, biomarker-based selection, and dynamic evolution of tumor-immune interactions. Patient enrollment increasingly relies on molecular and immunological profiling, including HLA type, neoantigen burden, TMB, and immune-infiltration signatures, to enrich for likely responders and minimize cohort heterogeneity. Additionally, manufacturing timelines must align with disease kinetics, necessitating rapid, automated production workflows to ensure timely vaccine delivery for patients with aggressive malignancies. Traditional response evaluation criteria in solid tumors (RECIST) often inadequately to capture the delayed or atypical response associated with immunotherapy [243], [244]. Incorporation of immune-related response criteria (irRC), minimal residual disease (MRD) monitoring, and antigen-specific T cell expansion metrics is essential is essential for accurate assessment [245].

Establishing appropriate control arms for highly individualized vaccines remains a major challenge. Adaptive trial schemas, including crossover designs, N-of 1 trials, or synthetic control arms, and Bayesian adaptive randomization, offer feasible strategies to generate statistically meaningful data while maintaining ethical and logistical feasibility [246], [247]. Looking ahead, basket or umbrella trials may further accelerate evaluation across tumor types or biomarker-defined subgroups, thereby improving scalability and regulatory efficiency.

Beyond trial architecture, AI is becoming integral to the design, optimization, and evaluation of biomimetic nanovaccines. AI-driven neoantigen prediction pipelines leverage deep learning on immunopeptidomics and MHC-binding datasets to improve selection of highly immunogenic epitopes, directly enhancing vaccine potency [248], [249]. Machine learning (ML) models trained on nanoparticle libraries can predict optimal physicochemical parameters including size, charge, lipid composition, and membrane-coating density to improve DC uptake, lymph-node trafficking, and controlled antigen release [250], [251]. AI-based immunodynamics models can simulate T-cell priming, exhaustion trajectories, and epitope spreading, informing rational combination strategies with ICB or innate agonists [252].

In manufacturing, computer vision and ML algorithms enable real-time quality control by detecting membrane-coating heterogeneity, aggregation states, or deviations in nanoparticle morphology [253]. Digital twins’ virtual replicas of the production pipeline can optimize flow parameters, microfluidic assembly, and batch scalability, supporting GMP compliance and reducing production variability [254]. Finally, AI-enhanced clinical analytics can correlate longitudinal biomarker profiles (e.g., circulating TCR clonotypes, cytokine signatures, spatial immune imaging) with treatment outcomes, providing adaptive feedback to refine dosing intervals, antigen selection, and patient stratification in ongoing trials [255], [256].

Overall, integrating AI across antigen discovery, formulation design, manufacturing, and clinical decision-support provides a continuous optimization loop that strengthens the translational trajectory of biomimetic nanovaccines and accelerates their clinical readiness.

## Conclusion

Nanovaccine platforms have made significant strides in overcoming long-standing barriers to effective cancer immunotherapy. As discussed throughout this review, biomimetic coatings such as RBC membranes, exosomes, and synthetic lipid bilayers represent a transformative approach for enhancing stability, immune targeting, and antigen delivery in mRNA- and peptide-based vaccines. These surface-engineered systems extend circulation, enhance lymph node trafficking, and facilitate robust DC-mediated antigen presentation, thereby amplifying adaptive immune responses.

In parallel, the emergence of personalized and autologous nanoparticle platforms incorporating patient-specific neoantigens or membrane components underscores a broader shift toward individualized precision immunotherapy. When combined with ICIs, PRR agonists, or radiotherapy, these nanovaccines demonstrate synergistic reprogramming of the tumor-immune microenvironment and durable antitumor activity in preclinical models.

Despite these advances, translational barriers remain. The mechanisms by which biomimetic coatings modulate innate and adaptive immunity are not fully understood, and the identification of predictive biomarkers to guide patient selection remains in its infancy. The incorporation of AI-driven antigen discovery and nanoparticle design is promising but requires standardized validation pipelines. Moreover, clinical trial frameworks must evolve to accommodate individualized production, adaptive endpoints, and real-world scalability.

Looking ahead, biomimetic and modular nanovaccine systems are poised to serve as foundational components of next-generation cancer immunotherapy. Their inherent adaptability and compatibility with combination strategies position them as powerful tools in the era of precision oncology. Realizing their full clinical potential will depend on interdisciplinary collaboration across immunology, materials science, computational biology, and regulatory science to bridge innovation with clinical implementation.

## Declaration of competing interest

The authors declare that they have no known competing financial interests or personal relationships that could have appeared to influence the work reported in this paper.
